# AI-Based OCT Assessment of Anatomic Response During the Loading Phase of Anti-VEGF Therapy in Neovascular Age-Related Macular Degeneration

**DOI:** 10.1167/iovs.67.12.62

**Published:** 2026-10-26

**Authors:** Marco Nassisi, Chiara Maria Eandi, Caterina Donà, Francesco Pozzo Giuffrida, Jennifer Cattaneo, Carlo Alessandro Lavia, Alessia Malerba, Davide Galli, Claudia Mainetti, Luca Gambaro, Francesco Faraldi, Laura Dell'Arti, Chiara Mapelli, Giuseppe Casalino, Francesco Viola

**Affiliations:** 1 Dipartimento di Scienze Cliniche e di Comunità, Dipartimento di Eccellenza 2023–2027, University of Milan, Milan, Italy; 2 Ophthalmological Unit, Fondazione IRCCS Ca’ Granda Ospedale Maggiore Policlinico, Milan, Italy; 3 Department of Ophthalmology, Jules-Gonin Eye Hospital, Fondation Asile des Aveugles, University of Lausanne, Lausanne, Switzerland; 4 Department of Surgical Sciences, University of Torino, Torino, Italy; 5 Ophthalmology Unit, Surgical Department, A.O. Ordine Mauriziano, Turin, Italy; 6 Ophthalmology Unit, Surgical Department, ASL TO5, Chieri, Italy

**Keywords:** neovascular age-related macular degeneration, anti-VEGF therapy, artificial intelligence, biomarkers, loading phase, retina

## Abstract

**Purpose:**

To compare functional and anatomic outcomes after the loading phase of intravitreal bevacizumab, aflibercept 2 mg, or faricimab in treatment-naive neovascular age-related macular degeneration (nAMD), using artificial intelligence (AI)–based optical coherence tomography (OCT) analysis.

**Methods:**

This multicenter retrospective cohort study included 224 eyes. Patients received three monthly injections of bevacizumab (*n* = 80), aflibercept 2 mg (*n* = 78), or faricimab (*n* = 66). Best-corrected visual acuity (BCVA) and spectral-domain OCT were assessed at baseline and week 12. AI-based segmentation quantified volumetric measurements of intraretinal fluid (IRFv), subretinal fluid (SRFv), and pigment epithelial detachment. The primary outcome was qualitative dry macula (absence of intraretinal fluid and subretinal fluid). An exploratory composite OCT biomarker (ΔCOMBO), defined as the lesser percentage reduction between IRFv and SRFv, was evaluated as a hypothesis-generating metric using receiver operating characteristic analysis. Baseline-adjusted analyses and multivariable logistic regression were performed.

**Results:**

Descriptive within-group analyses showed improvement in BCVA and reduction in OCT parameters at week 12 in treatment groups. BCVA improvement was statistically significant in the bevacizumab and faricimab groups. In baseline-adjusted analyses, between-group differences emerged for OCT outcomes, whereas no significant between-group differences were observed for BCVA. Higher rates of dry macula were observed with faricimab (80.3%) and aflibercept 2 mg (67.9%) compared with bevacizumab (41.3%) (*P* < 0.001). The exploratory composite metric ΔCOMBO showed high discriminative performance (area under the curve = 0.959; 95% confidence interval, 0.907–0.999).

**Conclusions:**

All agents improved functional and anatomic outcomes after the loading phase in treatment-naive nAMD. Baseline-adjusted analyses showed differences in anatomic response patterns across treatments. Exploratory AI-based OCT biomarkers may help characterize short-term anatomic response; however, these findings require external validation.

Neovascular age-related macular degeneration (nAMD) is the leading cause of severe vision impairment in individuals over 50 years of age, with prevalence expected to rise due to global population aging. The development of macular neovascularization leads to exudation, fibrosis, and atrophy, often resulting in irreversible central vision loss.^1^

Despite proven efficacy and safety, no anti-VEGF agent has demonstrated unequivocal superiority in randomized clinical trials. Head-to-head comparative evidence remains limited, and bevacizumab is widely used in real-world practice due to cost considerations,^2^^–^^9^ including its authorized use under Italian regulatory guidance (AIFA Note 98, December 31, 2020).^10^

Recent high-level comparative evidence further supports the relevance of evaluating differences among anti-VEGF agents in real-world settings.^11^ Newer agents may offer anatomic benefits beyond visual acuity gains. Post hoc analyses from the TENAYA and LUCERNE trials showed higher rates of fluid resolution with faricimab compared with aflibercept 2 mg at week 12, despite similar best-corrected visual acuity (BCVA) gains.^12^ These findings highlight a potential dissociation between functional and anatomic responses and underscore the relevance of early fluid control as a predictor of long-term outcomes.^13^^,^^14^ To date, no real-world study has directly compared bevacizumab, aflibercept 2 mg, and faricimab under equivalent clinical conditions in treatment-naive eyes.

Real-world monitoring of disease activity is variable, supporting the need for objective and reproducible optical coherence tomography (OCT)–based metrics.^15^^–^^17^ In this context, artificial intelligence (AI) offers a promising approach to enhance consistency in disease activity assessment through automated quantification of retinal fluid and lesion features.^18^ More broadly, recent evidence syntheses on AI in age-related macular degeneration (AMD) suggest substantial promise for imaging-based prediction, while also underscoring the need for external validation and methodological standardization.^19^

This study aimed to compare early functional and anatomic outcomes in treatment-naive nAMD eyes treated with bevacizumab, aflibercept 2 mg, or faricimab following a standardized three-injection loading regimen. An AI-based platform (Discovery platform, Discovery OCT Fluid and Biomarker Detector; RetinAI Medical AG, Bern, Switzerland) was used to quantify volumetric measurements of intraretinal fluid, subretinal fluid, and pigment epithelial detachment volumes (IRFv, SRFv, and PEDv, respectively) and to evaluate AI-derived definitions of disease activity against expert qualitative grading in a real-world setting.

## Methods

### Study Design and Ethical Approval

Data collection for this retrospective multicenter study was approved by the local institutional review boards of Fondazione IRCCS Ca’ Granda Ospedale Maggiore Policlinico di Milano (Milan, Italy), Azienda Ospedaliera Ordine Mauriziano di Torino SC Oculistica (Turin, Italy), and Hôpital Ophtalmique Jules-Gonin, Fondation Asile des Aveugles (Lausanne, Switzerland). The study adhered to the principles of the Declaration of Helsinki. Due to its retrospective nature, the institutional review boards granted a waiver of informed consent.

### Study Population

This multicentric retrospective study included naive patients with nAMD who started treatment with faricimab, aflibercept 2 mg, or bevacizumab injections between August 2019 and November 2023.

All patients received three consecutive monthly intravitreal injections and were evaluated at baseline and at week 12. Inclusion required a minimum follow-up of 3 months from treatment initiation. Exclusion criteria were high myopia (>−6 diopters), history of vitrectomy, or any vitreoretinal comorbidities and any treatment interruption or treatment switch during the 3-month follow-up period. Only one eye per patient was included in the analysis to ensure independence of observations. Treatment allocation reflected routine clinical practice at each participating center and was primarily influenced by local reimbursement policies, temporal drug availability, center-specific treatment patterns, and physician preference. Given the nonrandomized treatment allocation, all comparative analyses were interpreted as adjusted observational estimates rather than causal treatment effects. Residual confounding due to unmeasured variables cannot be excluded.

### Ophthalmologic Evaluation

All patients underwent a comprehensive ophthalmologic examination, including BCVA measurement using the Early Treatment Diabetic Retinopathy Study (ETDRS) chart (expressed in ETDRS letters) and spectral-domain OCT imaging with the Spectralis system (Heidelberg Engineering, Heidelberg, Germany) at baseline and each follow-up visit. OCT scans consisted of 49 B-scans covering at least 6 × 6 mm, with averaging of nine B-scans per horizontal scan; only scans with a signal strength ≥20 were included.

At baseline, fluorescein angiography and indocyanine green angiography were performed to classify neovascular AMD subtypes, including type 1, type 2, type 3 macular neovascularization, mixed (types 1+2), and polypoidal choroidal vasculopathy.

### Data Collection and Analysis

Clinical data were retrospectively collected from medical records, including demographics (age, gender), eye laterality, and BCVA at baseline and 1 month after the loading phase of three-monthly anti-VEGF injections (week 12).

High-resolution OCT volumes were anonymized and exported to the Discovery platform (RetinAI Medical AG), a Conformité Européenne (CE)-marked, AI-powered cloud-based solution for automated fluid and retinal layer segmentation. Quantitative parameters extracted included central retinal thickness (CRT), central choroidal thickness (CCT), IRFv, SRFv, and retinal PEDv at baseline and week 12. A representative example of AI-based OCT segmentation and quantitative fluid assessment is provided in Supplementary Figure S1. The underlying deep learning approach for fluid detection and volumetric quantification in neovascular AMD has been previously validated in a peer-reviewed study, which reported high detection performance, strong agreement with expert manual annotations, and high repeatability of volumetric measurements on repeated same-day OCT acquisitions.^20^ In the present study, the segmentation model was used “as is,” without any training or fine-tuning on the current dataset. A total of 232 potentially eligible patients were identified. Eight patients (3.4%) were excluded before the final analysis because their OCT volumes showed insufficient image quality or gross segmentation errors that precluded reliable quantitative assessment. Excluded patients were distributed across treatment groups (*n* = 3 bevacizumab, *n* = 3 aflibercept 2 mg, and *n* = 2 faricimab). The final analytic cohort therefore included 224 eyes from 224 patients. No systematic manual correction of automated segmentations was performed. Disease activity was assessed both qualitatively and quantitatively. Qualitative absence of activity (“dry macula”) was defined by the absence of IRFv and SRFv, in line with Moorfields anatomic criteria, which define qualitative dry macula as the absence of intraretinal and subretinal fluid on OCT, with any detectable retinal fluid considered indicative of active disease.^21^^,^^22^ Two independent retina specialists (CD and AM) graded disease activity. Intergrader agreement was high (Cohen's κ = 0.87). Disagreements were adjudicated by a senior grader (MN) to generate the reference label. Incidence of foveal-involving complete outer retinal and retinal pigment epithelium atrophy (cRORA) and/or fibrosis (defined as a highly reflective with well-demarcated, sharp borders and loss or disruption of overlying ellipsoid zone and external limiting membrane) was evaluated.^23^^–^^25^ For quantitative assessment, we computed the relative percentage change between baseline and postloading values for each imaging parameter. These included ΔPED% (percentage change in retinal pigment epithelium detachment volume), ΔIRF% (percentage change in intraretinal fluid volume), ΔSRF% (percentage change in subretinal fluid volume), ΔCRT% (percentage change in central retinal thickness), ΔCCT% (percentage change in central choroidal thickness), and an exploratory composite OCT biomarker (ΔCOMBO), reflecting the least favorable percentage reduction between ΔIRF% and ΔSRF% for each patient. This conservative definition was chosen because persistent fluid in either the intraretinal or subretinal compartment is generally considered indicative of ongoing disease activity in routine clinical practice. Using the least favorable percentage reduction therefore avoids masking residual activity that could occur with averaging or weighted composite measures while accounting for baseline differences in fluid burden. Qualitative grading was performed independently by two retina specialists, who were masked to AI-derived quantitative measurements, treatment assignment, and visit order. No calibration analysis, external validation, or reproducibility testing across independent cohorts was performed in the present dataset; accordingly, any biomarker thresholds derived here should be considered internal and exploratory.

### Statistical Analysis

Statistical analysis was performed using SPSS version 29.0.1.0 (IBM, Chicago, IL, USA). The primary outcome was qualitative dry macula at week 12, defined by expert grading as the absence of intraretinal and subretinal fluid. Secondary outcomes included change in BCVA and quantitative OCT-derived anatomic parameters, including CRT, CCT, IRFv, SRFv, and PEDv. Analyses evaluating associations between OCT-derived biomarkers and qualitative dry macula, including receiver operating characteristic (ROC) analysis, derivation of the ΔCOMBO threshold, and AI-derived dry macula classification, were considered exploratory. The main comparative analyses consisted of baseline-adjusted between-group analyses, whereas within-group comparisons and ROC-based biomarker analyses were considered descriptive or exploratory. Continuous variables were tested for normality using the Shapiro–Wilk test and are reported as mean ± SD or median and interquartile range, as appropriate. Within-group changes between baseline and week 12 were assessed using paired *t*-tests and are reported for descriptive purposes only. Primary between-group comparisons were conducted using baseline-adjusted analysis of covariance (ANCOVA), modeling week 12 values as dependent variables, treatment group as the main factor, and baseline values as covariates. Given their skewed distribution, intraretinal, subretinal, and pigment epithelial detachment volumes were log-transformed (ln[x + c]), while central retinal and choroidal thicknesses were analyzed without transformation. Covariates were prespecified based on clinical relevance and potential association with treatment allocation or anatomic response to partially account for baseline imbalance and center-level practice variation inherent to the observational study design. These covariates included age, baseline BCVA, lens status, macular neovascularization subtype, and retinal center. Treatment center was included to partially account for center-specific treatment patterns and practice variability. Pairwise comparisons were adjusted for multiple testing. These baseline-adjusted models constituted the primary comparative analyses of the study. Associations between OCT biomarkers and qualitative dry macula were evaluated using univariable logistic regression and ROC analysis, with optimal cutoffs derived using Youden's index. These analyses were considered exploratory.

Multivariable logistic regression models were constructed using qualitative dry macula as the dependent variable and a predefined adjustment set, including treatment, age, baseline BCVA, lens status, macular neovascularization subtype, and treatment center. A core multivariable model including only clinical covariates was first constructed. OCT-derived biomarkers (ΔIRF%, ΔSRF%, ΔCRT%, ΔPED%, ΔCCT%, and ΔCOMBO) were then added individually in separate models to avoid multicollinearity and to evaluate their incremental association with qualitative dry macula. Model fit was assessed using −2 log likelihood and Nagelkerke *R*², and collinearity was assessed using variance inflation factors. No propensity-based adjustment was performed. Accordingly, all adjusted models should be interpreted as observational association models rather than causal treatment effect estimates. Full multivariable model summaries and sensitivity analyses are reported in the Supplementary Material.

## Results

The study included 224 eyes from 224 patients: 80 treated with bevacizumab (35.7%), 78 with aflibercept 2 mg (34.8%), and 66 with faricimab (29.5%). As shown in Table 1, the three groups were well balanced in terms of age, sex, and baseline visual acuity. However, a significantly higher proportion of pseudophakic patients was observed in the aflibercept 2-mg and faricimab groups (*P* < 0.001). Between-group comparisons are based on baseline-adjusted analyses, while within-group changes are reported for descriptive purposes only. Baseline and week 12 values for all quantitative OCT biomarkers are presented in Table 2.

**Table 1.** Baseline Characteristics of the Study Population

Characteristic	Bevacizumab	Aflibercept 2 mg	Faricimab	*P* Value
No. of eyes	80	78	66	—
Age, mean (SD), y	79.1 (6.8)	80.17 (8.4)	79.3 (8.7)	0.658^*^
Female, *n* (%)	57 (70.4)	44 (56.4)	40 (60.6)	0.172^†^
Follow-up, mean (SD), wk	12.8 (1.2)	12.9 (1.3)	12.5 (1.2)	0.483^*^
Pseudophakia, *n* (%)	21 (26.9)	47 (60.3)	35 (53.0)	<0.001^†^
BCVA, ETDRS letters, mean (SD)	59.76 (16.61)	62.17 (20.74)	63 (15.21)	0.511^*^
MNV type, *n* (%)				0.226^‡^
Type 1	42 (51.9)	41 (52.7)	29 (43.9)	
Type 2	13 (16.1)	17 (21.8)	14 (21.2)	
Type 3	13 (16.1)	11 (14.1)	17 (25.8)	
PCV	6 (7.0)	4 (5.1)	6 (9.1)	
Mixed	7 (8.9)	5 (6.3)	0 (0)	
CRT, mean (SD), µm	495.6 (190.85)	458.73 (183.18)	461.88 (208.92)	0.420^*^
CCT, mean (SD), µm	190.67 (56.16)	171.08 (55.07)	188.71 (58.93)	0.063^*^

MNV, macular neovascularization; PCV, polypoidal choroidal vasculopathy.

* One-way ANOVA test.

† Fisher's exact test.

‡ Chi-squared test.

**Table 2.** Fluid Dynamics and Anatomic Response at 3 Months

	Bevacizumab			Aflibercept 2 mg			Faricimab				
	Baseline	Follow-Up	Within-Group *P* Value	Baseline	Follow-Up	Within-Group *P* Value	Baseline	Follow-Up	Within-Group *P* Value	Between-Groups *P* Value	Pairwise Summary^*^
CRT, mean (SD), µm	495.6 (190.85)	370.71 (153.36)	<0.001^†^	458.73 (183.18)	316.24 (122.21)	<0.001^†^	461.88 (208.92)	291.64 (80.58)	<0.001^†^	0.008^‡^	A: 0.076
											B: 0.002
											C: 0.863
CCT, mean (SD), µm	190.67 (56.16)	184.94 (56.89)	<0.001^†^	171.08 (55.07)	159.72 (58.15)	<0.001^†^	188.71 (58.93)	182.15 (62.19)	<0.001^†^	0.369^‡^	A: 1.000
											B: 1.000
											C: 1.000
PEDv, mean (SD), nL	880.45 (1149.71)	631.52 (887.12)	0.006^†^	675.40 (1465.36)	291.49 (439.51)	0.009^†^	741.77 (1170.88)	364.83 (682.78)	0.003^†^	<0.001^‡^	A: 0.120
											B: 0.003
											C: 0.808
SRFv, mean (SD), nL	489.84 (645.95)	76.33 (156.04)	<0.001^†^	427.51 (814.13)	8.05 (39.72)	<0.001^†^	466 (740.42)	43.62 (196.88)	<0.001^†^	<0.001^‡^	A: <0.001
											B: 0.006
											C: 0.668
IRFv, mean (SD), nL	131.57 (205.1)	27.89 (69.62)	<0.001^†^	115.14 (207.77)	10.52 (69.77)	<0.001^†^	108.39 (305.6)	2.68 (9.55)	<0.001^†^	0.001^‡^	A: <0.001
											B: 0.002
											C: 1.000

* Pairwise comparisons were adjusted for multiple testing; A: bevacizumab versus aflibercept 2 mg; B: bevacizumab versus faricimab; C: aflibercept 2 mg versus faricimab.

† Paired samples *t*-test.

‡ ANCOVA with week 12 values as dependent variables and baseline values as covariates. Fluid volumes were log-transformed (ln[x + c]) due to right-skewed distributions.

### Functional and Structural Outcomes

Descriptive within-group analyses showed improvement in BCVA at week 12 in all groups, but it reached statistical significance in the bevacizumab and faricimab groups. In the bevacizumab group, BCVA increased from 59.8 ± 16.6 to 62.3 ± 17.1 letters (mean change +2.5 ± 9.6; *P* = 0.022). In the aflibercept 2-mg group, BCVA improved from 62.2 ± 20.7 to 65.7 ± 18.9 letters (mean change +3.5 ± 11.0; *P* = 0.060), and in the faricimab group, BCVA increased from 63.0 ± 15.2 to 66.5 ± 17.0 letters (mean change +3.5 ± 16.1; *P* = 0.042). Despite these within-group improvements, no significant between-group differences were observed in adjusted comparisons (*P* = 0.511). All three agents produced a significant reduction in CRT from baseline to week 12. The mean CRT reduction was −129.5 ± 159.2 µm for bevacizumab, −142.5 ± 143.6 µm for aflibercept 2 mg, and −170.2 ± 183.5 µm for faricimab, with all changes reaching statistical significance (*P* < 0.01 for all). In contrast, baseline-adjusted analyses showed a significant difference between bevacizumab and faricimab (Table 2). As for choroidal thickness, bevacizumab induced a slightly smaller mean reduction (−6.2 ± 15.7 µm) than aflibercept 2 mg (−11.4 ± 17.9 µm) and faricimab (−6.6 ± 14.4 µm). While CCT change was statistically significant within each group (*P* < 0.01), the differences in CCT reduction among treatment groups were not statistically significant (Table 2). At week 12, no significant differences were observed among groups in the incidence of foveal-involving cRORA (2 cases with bevacizumab, 3 with aflibercept 2 mg, and 3 with faricimab; *P* = 0.807) or fibrosis (14 cases with bevacizumab, 6 with aflibercept 2 mg, and 8 with faricimab; *P* = 0.186). Retinal pigment epithelium tears occurred in two eyes treated with bevacizumab, one with aflibercept 2 mg, and three with faricimab (*P* = 0.476). No other ocular or systemic adverse events were reported during follow-up.

### Fluid Dynamics and Anatomic Response

Descriptive within-group analyses demonstrated a significant reduction in PEDv, SRFv, and IRFv in all treatment groups. PEDv decreased significantly from 880.5 to 631.5 nL with bevacizumab (*P* = 0.006), from 675.4 to 291.5 nL with aflibercept 2 mg (*P* = 0.009), and from 741.8 to 364.8 nL with faricimab (*P* = 0.003). SRFv similarly decreased in all groups, from 489.8 to 76.3 nL in bevacizumab-treated eyes, from 427.5 to 8.1 nL with aflibercept 2 mg, and from 466.0 to 43.6 nL with faricimab (*P* < 0.001 for all). IRFv reduction was statistically significant in all groups, decreasing from 131.6 to 27.9 nL in the bevacizumab group, from 115.1 to 10.5 nL in the aflibercept 2-mg group, and from 108.4 to 2.7 nL in the faricimab group (*P* < 0.001 for all comparisons). Adjusted analyses showed greater intraretinal and subretinal fluid reductions with aflibercept 2 mg and faricimab compared with bevacizumab, with no significant differences between the two on-label agents; in contrast, PEDv showed a significantly greater reduction with faricimab compared with bevacizumab, while no significant differences were observed between aflibercept 2 mg and the other treatment groups (Table 2).

### Exploratory Analyses of AI-Derived OCT Biomarkers

Within the exploratory biomarker analyses, ΔCOMBO showed the strongest association with qualitative dry macula after the loading phase and was therefore selected as the imaging predictor for the main analysis. The multivariable model including ΔCOMBO is reported in Table 3. In this adjusted analysis, ΔCOMBO remained independently associated with dry macula (adjusted odds ratio [OR], 1.48; 95% confidence interval [CI], 1.19–1.85; *P* < 0.001). After adjustment for anatomic response, treatment group was not independently associated with dry macula. Treatment effect estimates should nevertheless be interpreted cautiously, as the wide confidence intervals indicate limited precision after adjustment.

**Table 3.** Multivariable Logistic Regression for Qualitative Dry Macula

Variable	Adjusted OR	95% CI	*P* Value
ΔCOMBO	1.48	1.19–1.85	<0.001
Treatment: aflibercept vs. bevacizumab	1.00	0.04–24.26	0.999
Treatment: faricimab vs. bevacizumab	0.61	0.02–17.38	0.772
Age (per year)	0.98	0.85–1.14	0.825
Baseline BCVA (ETDRS letters)	0.93	0.86–1.02	0.112
Pseudophakia (yes vs. no)	2.25	0.24–21.39	0.479
MNV subtype (overall)	—	—	0.767
Retinal center (overall)	—	—	0.639

The multivariable model included 136 qualitative dry macula events among 224 eyes. ΔCOMBO was evaluated as the sole OCT-derived biomarker to avoid multicollinearity. Full model summaries and alternative biomarker models are reported in the Supplementary Material. ΔCOMBO: combined fluid variable, defined as the least favorable percent reduction between ΔIRF% and ΔSRF% for each subject.

Other OCT-derived biomarkers showed weaker or less consistent associations with qualitative dry macula and are reported in the Supplementary Material together with full model summaries and model fit comparisons (Supplementary Tables S1–S4). Compared with the core adjusted model, inclusion of ΔCOMBO resulted in the largest improvement in model fit, as reflected by likelihood-based metrics (Δ−2 log likelihood), indicating superior explanatory performance relative to other OCT-derived parameters (Supplementary Table S5).

Receiver operating characteristic analysis confirmed the discriminative performance of ΔCOMBO for qualitative dry macula (area under the curve = 0.959; 95% CI, 0.907–0.999; Fig.). Using Youden's index, the optimal ΔCOMBO threshold was identified at 99%. Sensitivity analyses using alternative thresholds yielded consistent between-group patterns (Supplementary Table S6).

**Figure.**
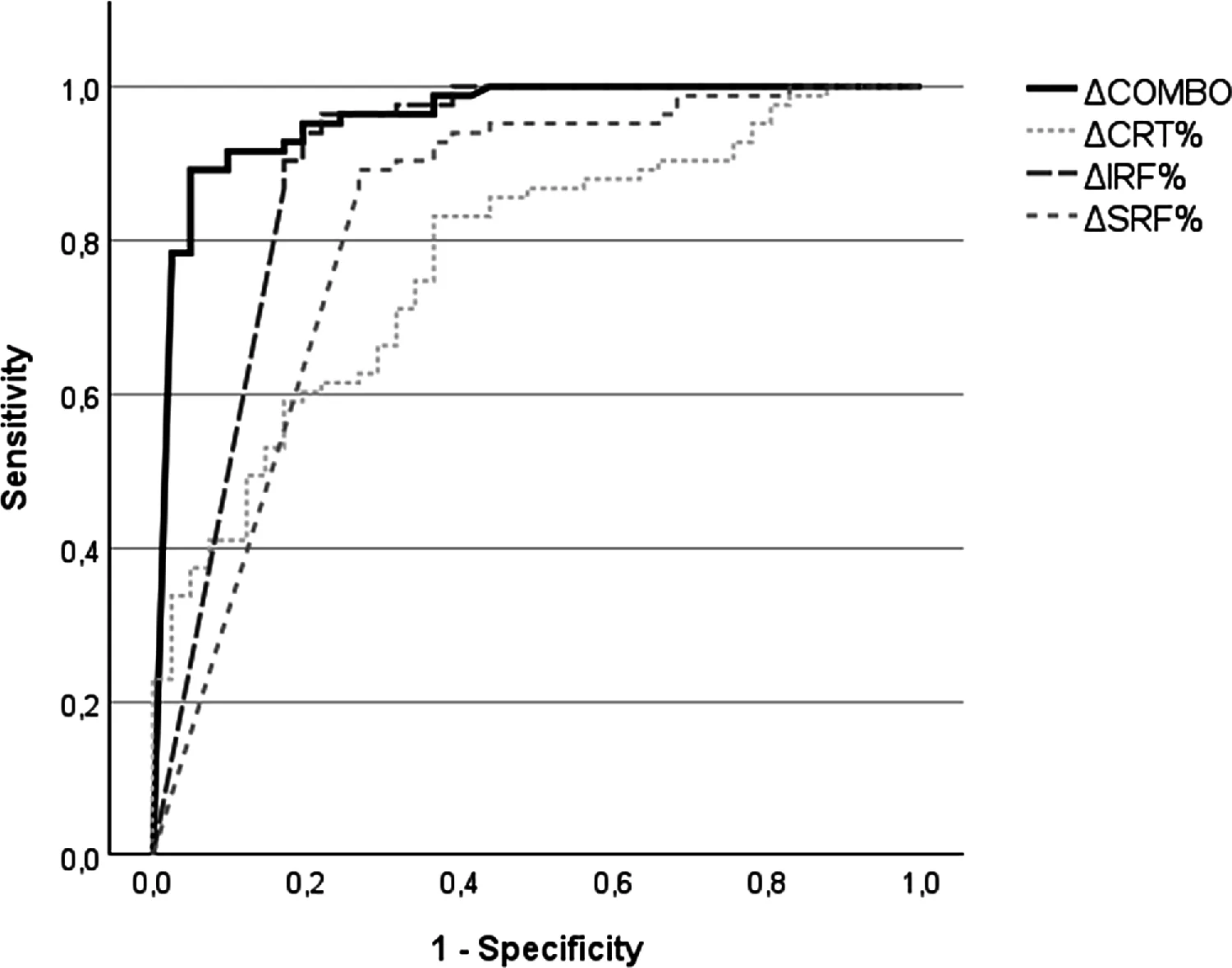
ROC curves for the prediction of clinical disease activity after the loading phase, based on percent change in SRFv (SRF%), IRFv (ΔIRF%), CRT (ΔCRT%), and the exploratory composite OCT biomarker (ΔCOMBO). The area under the curve (AUC) was highest for ΔCOMBO (AUC = 0.959; 95% CI, 0.907–0.999), indicating the highest discriminative performance among evaluated parameters.

### Disease Activity

In an exploratory quantitative classification based on the internally derived ΔCOMBO threshold, 41.25% (33/80) of eyes in the bevacizumab group, 67.94% (53/78) in the aflibercept 2-mg group, and 80.3% (53/66) in the faricimab group met the AI-derived dry macula criterion (*P* < 0.001). Both on-label treatments were associated with significantly higher dry macula rates compared with bevacizumab. Sensitivity analyses using alternative definitions of dry macula based on absolute residual fluid volumes (0–20 nL) yielded consistent between-group patterns and are reported in Supplementary Tables S7 and S8. Similarly, qualitative assessment by retinal specialists confirmed higher rates of dry macula in the aflibercept 2-mg (65.38%) and faricimab (74.24%) groups than in the bevacizumab group (45%) (*P* < 0.001).

## Discussion

In this multicenter real-world study, we evaluated short-term functional and anatomic outcomes after a three-injection loading phase with bevacizumab, aflibercept 2 mg, or faricimab in treatment-naive nAMD, using AI-based OCT fluid quantification. The principal finding is that, although descriptive within-group improvements were observed across all treatments, baseline-adjusted analyses consistently demonstrated differences in anatomic response patterns. Accordingly, all between-group interpretations in this study are based on adjusted analyses, while unadjusted within-group changes are reported for descriptive purposes only.

Across baseline-adjusted analyses, aflibercept 2 mg and faricimab were associated with greater reductions in intraretinal and subretinal fluid compared with bevacizumab, with no significant differences between the two on-label agents. Similarly, pigment epithelial detachment volume showed a significantly greater reduction with faricimab compared with bevacizumab, whereas central choroidal thickness did not differ between groups. The selective emergence of between-group differences for OCT parameters directly related to exudative disease activity, but not for choroidal thickness, supports the biological plausibility of these findings.

These baseline-adjusted continuous anatomic results were consistent with the qualitative assessment of disease activity. Consistent with the primary comparative analyses, using the AI-derived ΔCOMBO, dry macula was achieved more frequently with faricimab and aflibercept 2 mg than with bevacizumab. When OCT-derived biomarkers were included in multivariable models, treatment group was no longer independently associated with the binary dry macula endpoint, and treatment effect estimates were attenuated after inclusion of anatomic response metrics. ΔCOMBO showed the strongest association with qualitative dry macula within this dataset and provided the largest improvement in model fit. However, this composite metric should be considered exploratory. It should therefore be regarded as a hypothesis-generating imaging biomarker intended to support future prospective validation studies rather than immediate clinical implementation. No calibration analysis, reproducibility assessment across independent cohorts, or external validation was performed, and the internally derived threshold should not be interpreted as clinically transferable without confirmation in external datasets. Furthermore, as ΔCOMBO relies on quantitative fluid measurements, its application requires access to validated segmentation tools and may not be directly transferable across different platforms without external validation.

The consistency of findings across baseline-adjusted continuous OCT parameters, AI-derived composite metrics, and qualitative grading strengthens the robustness of the observed anatomic pattern during the loading phase. These results are partially aligned with post hoc analyses from the TENAYA and LUCERNE trials, which reported greater anatomic drying with faricimab compared with aflibercept 2 mg after the loading phase, despite similar functional outcomes.^12^ Similar trends have been reported in smaller real-world cohorts.^26^^,^^27^

From a clinical perspective, these findings may inform treatment adaptation in real-world settings, where bevacizumab is often initiated as first-line therapy due to cost considerations and subsequent decisions are guided primarily by anatomic response. Sensitive anatomic biomarkers—particularly when quantified using AI-based tools—may assist clinicians in identifying suboptimal anatomic control during the loading phase, supporting individualized treatment decisions within treat-and-extend paradigms. These findings should be interpreted cautiously given the observational design and the exploratory nature of the biomarker analyses. AI-derived anatomic metrics may complement clinical assessment, but the proposed thresholds require external validation before any potential clinical application.

Beyond anatomic response, treatment burden and adherence represent key considerations in the real-world management of nAMD. Frequent intravitreal injections and monitoring visits place a substantial burden on patients and caregivers and may negatively affect long-term adherence, particularly in elderly populations.^28^ Although the present study was limited to the loading phase and did not assess treatment intervals or adherence outcomes, the observed differences in early anatomic drying patterns may be clinically relevant in this context. In routine practice, more complete early anatomic control is often considered a prerequisite for interval extension within treat-and-extend regimens, which may in turn reduce visit frequency and caregiver burden. These considerations remain speculative within the scope of the current study and warrant prospective evaluation in longer-term cohorts.

This study has limitations. Its retrospective design and short follow-up limited to the loading phase preclude conclusions regarding long-term functional outcomes or durability of treatment effects. Treatment allocation was not randomized and reflected real-world practice, potentially introducing residual confounding despite multivariable adjustment. ANCOVA and multivariable adjustment may reduce measured baseline imbalance but cannot eliminate residual confounding by indication or unmeasured center- and time-related factors inherent to observational treatment allocation. Therefore, observed between-group differences should be interpreted as adjusted associations rather than estimates of comparative treatment efficacy. The ΔCOMBO threshold was internally derived and should be considered exploratory; external validation in independent datasets is warranted. Finally, although AI-based segmentation has demonstrated high accuracy and repeatability in prior studies,^18^ OCT volumes with insufficient image quality were excluded from quantitative analyses, and no manual correction was performed. Baseline differences in macular neovascularization subtype distribution across treatment groups may have influenced anatomic response despite adjustment and represent a potential source of residual confounding. We did not perform propensity-based sensitivity analyses or inverse probability weighting approaches, which should be considered in future confirmatory studies. Another limitation of our study is the use of aflibercept 2 mg, which—although still widely adopted—is being increasingly supplemented or replaced by the higher-dose formulation (aflibercept 8 mg), especially in patients with high disease burden or to enable extended dosing intervals.^29^^–^^31^ As such, our results may not fully reflect the evolving treatment landscape in which aflibercept 8 mg has become available and is showing promise in terms of durability and fluid control. However, it is also worth noting that this is the era in which biosimilar formulations are entering the market, including a biosimilar version of aflibercept 2 mg. Given their anticipated cost-effectiveness and accessibility, these biosimilars are likely to be broadly adopted, particularly as first-line agents in cost-conscious health care systems. Thus, our findings remain clinically relevant for informing initial therapeutic strategies in real-world settings where economic considerations play a major role.^32^ In conclusion, faricimab and aflibercept 2 mg showed greater short-term anatomic drying than bevacizumab during the loading phase, whereas short-term visual outcomes were comparable across treatments. AI-based OCT biomarkers may help characterize early anatomic response; however, these findings are exploratory, should not be interpreted as evidence of clinical superiority, and require external validation before clinical application.

## Supplementary Material

Supplement 1
