# Supplementary material for: AI-Based OCT Assessment of Anatomic Response During the Loading Phase of Anti-VEGF Therapy in Neovascular Age-Related Macular Degeneration
Source: Invest Ophthalmol Vis Sci. 2026 Oct 26;67(12):62. doi: 10.1167/iovs.67.12.62 (PMC13722965; doi:10.1167/iovs.67.12.62)
Supplement: Supplementary file 1 [file iovs-67-12-62_s001.docx]

**AI-based OCT assessment of anatomical response during the loading phase of anti-VEGF therapy in neovascular age-related macular degeneration**

**Marco Nassisi**^1,2,^***, Chiara Maria Eandi**^3,4,^***, Caterina Donà**^1^**, Francesco Pozzo Giuffrida**^1^**, Jennifer Cattaneo**^3^**, Carlo Alessandro Lavia**^5,6^**, Alessia Malerba**^1^**, Davide Galli**^2^**, Claudia Mainetti**^2^**, Luca Gambaro**^1^**, Francesco Faraldi**^5,6^**, Laura Dell’Arti**^2^**, Chiara Mapelli**^2^**, Giuseppe Casalino**^2^**, Francesco Viola**^1,2^

^1^ Dipartimento di Scienze Cliniche e di Comunità, Dipartimento di Eccellenza 2023-2027, University of Milan, Milan, Italy.

^2^ Ophthalmological Unit, Fondazione IRCCS Ca’ Granda Ospedale Maggiore Policlinico, Milan, Italy.

^3^ Department of Ophthalmology, Jules-Gonin Eye Hospital, Fondation Asile des Aveugles, University of Lausanne, Lausanne, Switzerland.

^4^ Department of Surgical Sciences, University of Torino, Torino, Italy.

^5^ Ophthalmology Unit, Surgical Department, A.O. Ordine Mauriziano, Turin, Italy.

^6^ Ophthalmology Unit, Surgical Department, ASL TO5, Chieri, Italy.

* These authors contributed equally to this work

**Corresponding Author:** Francesco Viola, MD, Dipartimento di Scienze Cliniche e di Comunità, Dipartimento di Eccellenza 2023-2027, University of Milan, Via Festa Del Perdono 17, 20122 Milan, Italy. ([francesco.viola@unimi.it](mailto:francesco.viola@unimi.it)).

**SUPPLEMENTARY MATERIAL - LIST**

**Supplementary Table 1.** Multivariable logistic regression for qualitative dry macula – core adjustment model (no optical coherence tomography biomarkers)

**Supplementary Table 2.** Multivariable logistic regression for qualitative dry macula – core model + ΔIRF%

**Supplementary Table 3.** Multivariable logistic regression for qualitative dry macula – core model + ΔSRF%

**Supplementary Table 4.** Multivariable logistic regression for qualitative dry macula – core model + other OCT biomarkers

**Supplementary Table 5.** Model fit comparison with the core adjusted model.

**Supplementary Table 6.** Sensitivity analysis for different ΔCOMBO thresholds

**Supplementary Table 7.** Dry macula rates based on absolute residual fluid volume thresholds (0, 10, and 20 nL).

**Supplementary Table 8.** Adjusted logistic regression for dry macula defined by absolute residual fluid volumes.

**Supplementary Figure 1.** Representative example of AI-based OCT segmentation in a treatment-naïve nAMD eye

**Supplementary Table 1. Multivariable logistic regression for qualitative dry macula – core adjustment model (no optical coherence tomography biomarkers)**

| **Variable** | **Adjusted OR** | **95% CI** | **P-value** |
| --- | --- | --- | --- |
| Treatment (overall) | — | — | <0.001 |
| Age (per year) | 0.98 | 0.95 – 1.02 | 0.385 |
| Baseline BCVA (ETDRS letters) | 1.00 | 0.98 – 1.02 | 0.937 |
| Pseudophakia (yes vs no) | 1.05 | 0.54 – 2.05 | 0.878 |
| MNV subtype (overall) | — | — | 0.364 |
| Retinal Center (overall) | — | — | 0.380 |

OR: odds ratio; CI: confidence interval; BCVA: best corrected visual acuity; MNV: macular neovascularization.

**Supplementary Table 2. Multivariable logistic regression for qualitative dry macula – core model +** ΔIRF%

| **Variable** | **Adjusted OR** | **95% CI** | **P-value** |
| --- | --- | --- | --- |
| ΔIRF% | 1.36 | 1.11 – 1.68 | 0.004 |
| Treatment (overall) | — | — | 0.157 |
| Age (per year) | 0.99 | 0.87 – 1.13 | 0.881 |
| Baseline BCVA (ETDRS letters) | 0.95 | 0.88 – 1.02 | 0.141 |
| Pseudophakia (yes vs no) | 1.81 | 0.25 – 13.23 | 0.558 |
| MNV subtype (overall) | — | — | 0.542 |
| Retinal Center (overall) | — | — | 0.500 |

OR: odds ratio; CI: confidence interval; ΔIRF%: percent change of intraretinal fluid; BCVA: best corrected visual acuity; MNV: macular neovascularization.

**Supplementary Table 3. Multivariable logistic regression for qualitative dry macula – core model +** ΔSRF%

| **Variable** | **Adjusted OR** | **95% CI** | **P-value** |
| --- | --- | --- | --- |
| ΔSRF% | 1.16 | 1.07 – 1.26 | <0.001 |
| Treatment (overall) | — | — | 0.088 |
| Age (per year) | 0.99 | 0.94 – 1.04 | 0.672 |
| Baseline BCVA (ETDRS letters) | 0.99 | 0.96 – 1.02 | 0.360 |
| Pseudophakia (yes vs no) | 1.68 | 0.64 – 4.39 | 0.290 |
| MNV subtype (overall) | — | — | 0.638 |
| Retinal Center (overall) | — | — | 0.381 |

OR: odds ratio; CI: confidence interval; ΔSRF%: percent change of subretinal fluid; BCVA: best corrected visual acuity; MNV: macular neovascularization.

**Supplementary Table 4. Multivariable logistic regression for qualitative dry macula – core model + other OCT biomarkers**

| **Variable** | **Adjusted OR** | **95% CI** | **P-value** |
| --- | --- | --- | --- |
| ΔCRT% | 1.03 | 1.01 – 1.05 | 0.001 |
| ΔPED% | 1.00 | 0.99 – 1.00 | 0.239 |
| ΔCCT% | 1.02 | 0.99 – 1.05 | 0.196 |

OR: odds ratio; CI: confidence interval; ΔCRT%: percent change of central retinal thickness; ΔPED%: percent change of retinal pigment epithelium detachment; ΔCCT%: percent change of central choroidal thickness.

**Supplementary Table 5. Model fit comparison with the core adjusted model.**

| **Model** | **-2 Log Likelihood** | **Δ(-2LL) vs Core** | **Nagelkerke R^2^** |
| --- | --- | --- | --- |
| Core model | 244.84 | — | 0.17 |
| Core + ΔCOMBO | 34.98 | 209.86 | 0.93 |
| Core + ΔIRF% | 39.21 | 205.63 | 0.86 |
| Core + ΔSRF% | 136.92 | 107.92 | 0.58 |
| Core + ΔCRT% | 232.46 | 12.38 | 0.24 |
| Core + ΔPED% | 235.65 | 9.19 | 0.19 |
| Core + ΔCCT% | 243.03 | 1.81 | 0.18 |

Δ(−2LL) values represent improvements in model fit relative to the core adjusted model. Each OCT-derived biomarker was evaluated in a separate model to avoid multicollinearity. ΔCOMBO: combined fluid variable, defined as the least favorable percent change between ΔIRF% and ΔSRF% for each subject; ΔCRT%: percent change of central retinal thickness; ΔPED%: percent change of retinal pigment epithelium detachment; ΔCCT%: percent change of central choroidal thickness; ΔSRF%: percent change of subretinal fluid; ΔIRF%: percent change of intraretinal fluid,

**Supplementary Table 6. Sensitivity analysis for different ΔCOMBO thresholds**

|  |  | **≥99%** | **≥95%** | **≥90%** |
| --- | --- | --- | --- | --- |
| Patients with dry macula | Bevacizumab | 33/80 | 37/80 | 43/80 |
|  | Aflibercept 2 mg | 53/78 | 55/78 | 61/78 |
|  | Faricimab | 53/66 | 55/66 | 59/66 |
| P value* |  | <0.001 | <0.001 | <0.001 |

*Chi-squared test; ΔCOMBO: combined fluid variable, defined as the least favorable percent change between ΔIRF% and ΔSRF% for each subject.

**Supplementary Table 7. Dry macula rates based on absolute residual fluid volume thresholds (0, 10, and 20 nL).**

| **Threshold** | **Bevacizumab** | **Aflibercept 2 mg** | **Faricimab** | **P-Value*** |
| --- | --- | --- | --- | --- |
| 0 nL | 25/80 | 54/78 | 44/66 | <0.001 |
| 10 nL | 40/80 | 68/78 | 54/66 | <0.001 |
| 20 nL | 43/80 | 73/78 | 55/66 | <0.001 |

*Chi-squared test.

**Supplementary Table 8. Adjusted logistic regression for dry macula defined by absolute residual fluid volumes.**

|  | **0 nL** | | **10 nL** | | **20 nL** | |
| --- | --- | --- | --- | --- | --- | --- |
| **Variable** | **OR**  **(95% CI)** | **p-value** | **OR**  **(95% CI)** | **p-value** | **OR**  **(95% CI)** | **p-value** |
| Aflibercept 2 mg vs Bevacizumab | 3.272  (1.377-7.776) | 0.007 | 6.306  (2.066-19.251) | 0.001 | 6.333  (1.957-20.491) | 0.002 |
| Faricimab vs Bevacizumab | 6.295  (2.890-13.713) | <0.001 | 15.154  (5.188-44.260) | <0.001 | 16.888  (5.262-54.199) | <0.001 |
| Age (per year) | 1.002  (0.973-1.032) | 0.887 | 1.012  (0.976-1.048) | 0.519 | 1.008  (0.969-1.050) | 0.679 |
| Baseline BCVA (ETDRS letters) | 1.000  (0.983-1.017) | 0.988 | 1.003  (0.983-1.023) | 0.769 | 0.992  (0.970-1.014) | 0.463 |
| Pseudophakia (yes vs no) | 0.864  (0.476-1.568) | 0.631 | 1.325  (0.646-2.716) | 0.443 | 1.252  (0.591-2.652) | 0.558 |
| MNV subtype (overall) | -- | 0.699 | -- | 0.793 | -- | 0.941 |
| Retinal Center (overall) | -- | 0.305 | -- | 0.250 | -- | 0.610 |

Dry macula was defined as residual intraretinal and subretinal fluid volumes equal to 0 nL, <10 nL, or <20 nL. Each model was adjusted for the same covariates. Results are reported as odds ratios (OR) with 95% confidence intervals (CI). BCVA: best corrected visual acuity; MNV: macular neovascularization.

**Supplementary Figure 1.** Representative example of AI-based OCT segmentation in a treatment-naïve nAMD eye at baseline (A) and after the loading phase (B). Intraretinal fluid (IRF, green), subretinal fluid (SRF, light blue), and pigment epithelial detachment (PED, dark blue) are automatically segmented and quantified. This example illustrates compartment-specific changes in retinal fluid following treatment and highlights the ability of AI-based segmentation to provide objective quantitative assessment of anatomical response.
